# Impact of Amarogentin on Gastric Carcinoma Cell Multiplication, Apoptosis and Migration via circKIF4A/miR-152-3p

**DOI:** 10.1155/2022/2156204

**Published:** 2022-06-14

**Authors:** Zhi Tan, Weining Wang, Jin Peng, Zhen Zhou, Jia Pan, Aiming Peng, Hui Cao, Wenling Fan

**Affiliations:** ^1^Department of Gastroenterology, First Hospital of Changsha, Changsha, 410005 Hunan, China; ^2^Department of Health Care, First Hospital of Changsha, Changsha, 410005 Hunan, China

## Abstract

**Objective:**

The active ingredients extracted from natural plants have anti-GC actions and can slow down gastric carcinoma (GC) progression. To investigate the impact of Amarogentin (AG) on GC cell multiplication, apoptosis and migration and the possible mechanisms.

**Methods:**

qRT-PCR quantification of circKIF4A and miR-152-3p in GC tissues and normal counterparts as well as HGC-27 (human GC cell strain) and GES-1 (human gastric mucosal epithelial cell strain) was performed. HGC-27 cells were intervened by AG of various concentrations. si-NC, si-circKIF4A were further transfected into HGC-27 cells. Besides, pcDNA and pcDNA-circKIF4A were transfected into HGC-27 cells, after which 60 mmol/L AG was added for intervention. Cell multiplication, clone formation, as well as apoptosis and migration measurements were made by MTT, plate clone formation, flow cytometry and Transwell assays, respectively; Double luciferase reporter assay was performed for targeting relationship identification between circKIF4A and miR-152-3p; Western blots were carried out to measure Bax and Bcl-2 protein levels.

**Results:**

circKIF4A increased (P <0.05) and miR-152-3p decreased (P <0.05) in GC tissues and cell strains. Concentration-dependently, AG intervention contributed to enhanced cell multiplication inhibitory rate, apoptosis rate, miR-152-3p expression and Bax protein level (P <0.05), together with declined number of cell clones formed, migrating cells, circKIF4A expression and Bcl-2 protein level (P <0.05). After transfection of si-circKIF4A, cell multiplication inhibition rate, apoptosis rate and Bax protein level enhanced (P <0.05), while cell clones formed and migrating cells as well as Bcl-2 protein level reduced (P <0.05). miR-152-3p can be controlled by circKIF4A; pcDNA-circKIF4A transfection antagonized AG's effects on HGC-27 cell multiplication, clone formation, apoptosis and migration.

**Conclusion:**

AG can decrease GC multiplication, clone formation and migration and induce apoptosis via modulating circKIF4A/miR-152-3p expression.

## 1. Introduction

In China, gastric carcinoma (GC) is a malignancy with high prevalence and death toll [1]. In the past few decades, there has been a worldwide trend of rapid urbanization and the adoption of westernized diet [2]. The previous global analysis of colorectal cancer incidence rate and mortality in 39 countries found that the incidence rate of young people (<50 years old) is significantly higher than that of people aged over 50 [3]. Advanced GC was found in most patients once diagnosed, attributing to the relatively hidden early clinical symptoms of the disease. Surgery and chemoradiotherapy are the mainstays to treat GC, but with unsatisfactory treatment outcomes and poor patient prognosis [4, 5].

Previous studies have demonstrated the long-term use of Chinese traditional herbal medicines in treating malignant diseases [6, 7]. The active ingredients extracted from natural plants have anti-GC actions and can slow down GC progression [8, 9]. Amarogentin (AG), first reported to exert anticarcinogenic action against cutaneous carcinoma via inducing cancer cell apoptosis, is the main active component isolated from Swertiamarin, which has been shown to promote liver cancer cell apoptosis and play an anti-liver cancer part [10–12]. However, the connection between AG and GC has been rarely reported. Except from this, circular RNAs (circRNAs) are ncRNA molecules with no 5'-end cap structure and 3'-end polyadenylate tail structure, which show aberrant expression in tumors and serve as potential targets for tumor targeted therapies. Reportedly, circRNAs interfere with cancer progression [13] and can be biomarkers to assist in cancer diagnosis and prognosis prediction [14]. Of them, circKIF4A is indicated to be up-regulated in triple negative breast cancer and can promote cancer development [15]. Moreover, following the binding to miRNA by MREs, circRNAs can be served as the regulatory gene for miRNAs [16, 17]. Starbase prediction showed that circKIF4A had binding sites with miR-152-3p, a gene that is down-regulated in GC cells and could inhibit GC cell multiplication and metastasis via up-regulating its expression [18].

However, the association between circRNAs and AG has not been reported in any kinds of cancer. Meanwhile, it remains unknown regarding the mechanism of circKIF4A/miR-152-3p axis in GC pathogenesis and development. Accordingly, the motivation and novelty of this research project is to investigate whether AG could affect GC multiplication, apoptosis and migration via modulating circKIF4A/miR-152-3p expression.

## 2. Data and Methods

### 2.1. Materials and Reagents

The specimens of cancer tissues and paracancerous counterparts of 57 GC cases visited and treated during the period of January 2020 and April 2020 were collected and stored in a cryogenic refrigerator at -80°C. Composed of 37 males and 20 females, all patients were pathologically diagnosed as GC, with an age ranging from 50 to 66 years (mean: 58.32 ± 4.16). Informed consent of patients or their close relatives was obtained, and this study met the relevant requirements of World Medical Association Declaration of Helsinki.

AG (Chengdu Alfa Biotech); Human GC cells HGC-27 and human gastric mucosal epithelial cellsGES-1 (ATCC); DMEM, trypsin, and fetal bovine serum (FBS) (Shanghai Beyotime Biotech); Trizol reagent and Lipofectamine™ 3000 Transfection Reagent (Invitrogen, USA); Reverse Transcription and Fluorescence Quantitative PCR (Thermo Fisher, USA); si-NC, si-circKIF4A, miR-NC, and miR-152-3p mimics (Guangzhou Ruibo Bio); pcDNA, pcDNA-circKIF4A (Shanghai Genomeditech); MTT reagent, apoptosis detection kit and Transwell chamber (Beijing Solarbio); Double luciferase reporter gene vector and its activity detection kit (Promega, USA); Rabbit anti-human Bax, Bcl-2 antibodies and HRP labeled goat anti-rabbit IgG secondary antibody (CST, USA).

### 2.2. Experimental Grouping

After seeding HGC-27 cells onto the wells of 6-well plates (1 × 10^5^ cells/well), they were cultivated in DMEM comprising AG of various concentrations (15, 30, and 60 mmol/L) for 24 h [19] and grouped as AG-L, -M and -H groups, respectively. Normal cultured HGC-27 cells were recorded as control group. Lipofectamine™ 3000 was used for the transfection. si-NC and si-circKIF4A groups were established by transfecting si-NC and si-circKIF4A into HGC-27 cells, respectively. Also, pcDNA and pcDNA-circKIF4A were transfected into HGC-27 cells. Following successful operation, they were cultured in DMEM with 60 mmol/L AG for 24 h and set as AG-H + pcDNA and AG-H + pcDNA-circKIF4A groups, respectively.

### 2.3. circKIF4A And miR-152-3p Detection by qRT-PCR

GC tissues, adjacent counterparts, as well as GES-1 and HGC-27 cells were added with 1 ml Trizol reagent to extract total RNA, after which cDNA synthesis was conducted by reverse transcription. The volume of qRT-PCR amplification reaction system was 20 *μ*L, comprising 10 *μ*L SYBR Green Master Mix, 0.8 *μ*L forward primers, 2 *μ*L cDNA, 0.8 *μ*L reverse primers, and ddH2O. The mixture was pre-denaturated (95°C/2 min), denaturated (95°C/15 s), annealed (60°C/30 s) and extended (72°C/30 s) for 40 cycles. Levels of circKIF4A relative to GAPDH and miR-152-3p to U6 were obtained via the 2^-*ΔΔ*Ct^ method. See Table 1 for primers used.

### 2.4. MTT Detection of Cell Multiplication

After planting HGC-27 cells into the wells of 96-well plates (3 × 10^3^ cells/well) and adding 20 *μ*L MTT solution to each well, cell culture was carried out in a 5%CO_2_ and 37°C incubator for 4 h to discard the supernatants. Following the addition of 150 *μ*L DMSO into each well, cells were cultivated (5 min) at indoor temperature without light. The came the detection of each well's absorbance (A)_490 nm_ with a microplate reader (Bio-Tek, Vermont, USA) and cell multiplication inhibition rate calculation: [(A of control group - A of experimental group)/(A of control group - A of blank group) ×100%].

### 2.5. Plate Clone Formation Experiment

In each group, HGC-27 cells were cultured in the incubator for 14 days after inoculating them into six-well plates (1 × 10^3^/well), with the culture medium changed every other day. After discarding the culture medium and rinsing with precooled PBS, the cells were processed for 500 *μ*L 4% paraformaldehyde immobilization (20 min), 400 *μ*L 1% crystal violet staining (15 min), distilled water rinsing and drying, for the final microscopic (Olympus, Tokyo, Japan) counting of the number of cell clones. Five randomly chosen fields were taken by microscope to count the number of colonies with more than 10 cells, and the mean was taken.

### 2.6. Flow Cytometry Detection of Apoptosis Rate

The HGC-27 cells collected from each group were subjected to 0.25% trypsin digestion and centrifugation (3000 r/min, 6 min) to collect cell precipitate for subsequent rising with precooled PBS, re-suspension with 500 *μ*L binding buffer, and incubation with AnnexinV-FITC and PI (5 *μ*L each, 10 min). Apoptosis rate determination was made by FACS Calibur flow cytometry (FACSCalibur, BD Biosciences).

### 2.7. Transwell Assay for Cell Migration

HGC-27 cells were gathered for inoculation in the apical chamber (1 × 10^5^ cells/well), and 600 *μ*L culture solution +10% FBS was placed into the basolateral one. After 24 h of cultivation, the cells were treated with PBS washing, paraformaldehyde fixation (20 min), and 0.1% crystal violet dyeing (10 min), for microscopical observation of migrating cells.

### 2.8. Detection of the Targeting Relationship between circKIF4Aand miR-152-3p via Double Luciferase Reporter Assay

The molecular cloning method was utilized to clone the binding loci of circKIF4A and miR-152-3p into pGL3 plasmid to build a wild-type vector WT-circKIF4A. Besides, the binding loci were mutated by gene mutation technology to construct a mutant vector MUT-circKIF4A containing the mutation site. Using lipofection, WT-circKIF4A and MUT-circKIF4A were simultaneously transfected into HGC-27 with miR-NC or miR-152-3p mimics, respectively, and then placed in an incubator for 48 h of further culture. Cells' luciferase activity was measured after cell collection.

### 2.9. Western Blotting of Bax and Bcl-2 Protein Expression

HGC-27 from each group were gathered and immersed in 500 *μ*L RIPA lysate for total protein isolation, after which the BCA method was utilized to quantify its concentration. 40 *μ*g protein specimens were subjected to SDS-PAGE, membrane transfer, and 2 h of sealing with 5% defatted milk. This was followed by culture (4°C, 24 hours) with Bax (1 : 1000), Bcl-2 (1 : 1000) primary antibodies and internal reference GAPDH antibody (1 : 30) diluent, as well as the subsequent incubation (37°C, 2 hours) with secondary antibody diluent (1 : 5000). Protein bands were quantified using Quantity One software.

### 2.10. Statistical Processing

Data analysis adopted SPSS21.0 (IBM, Chicago, IL) and P <0.05 was the threshold of significance. Quantitative data were expressed by (^−^x ± s), and the differences between groups and among multiple groups used independent samples t test and one-way ANOVA plus Bonferroni post hoc test, respectively.

## 3. Results

### 3.1. circKIF4A And miR-152-3p Expression in GC and Cells

**Figure 1:**
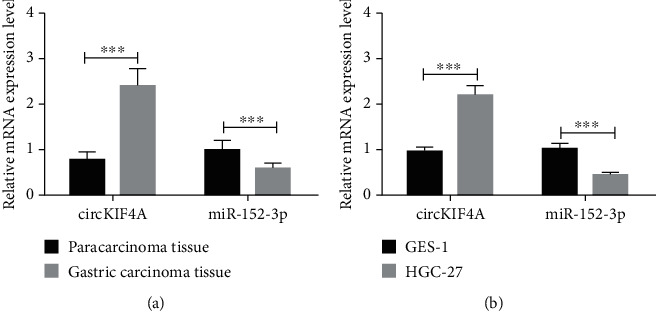
circKIF4A and miR-152-3p levels. a: circKIF4A and miR-152-3p expression in GC. b: circKIF4A and miR-152-3p expression in GC cells. ∗∗∗ P <0.001.

Compared with adjacent counterparts, circKIF4A increased in GC tissues (P <0.05) while miR-152-3p decreased (P <0.05), as shown in Figure 1(a); Similarly, elevated circKIF4A and reduced miR-152-3p were found in HGC-27 cells (P <0.05), versus GES-1 cells, as shown in Figure 1(b).

### 3.2. Impact of AG on HGC-27 Multiplication, Migration and Apoptosis

**Figure 2:**
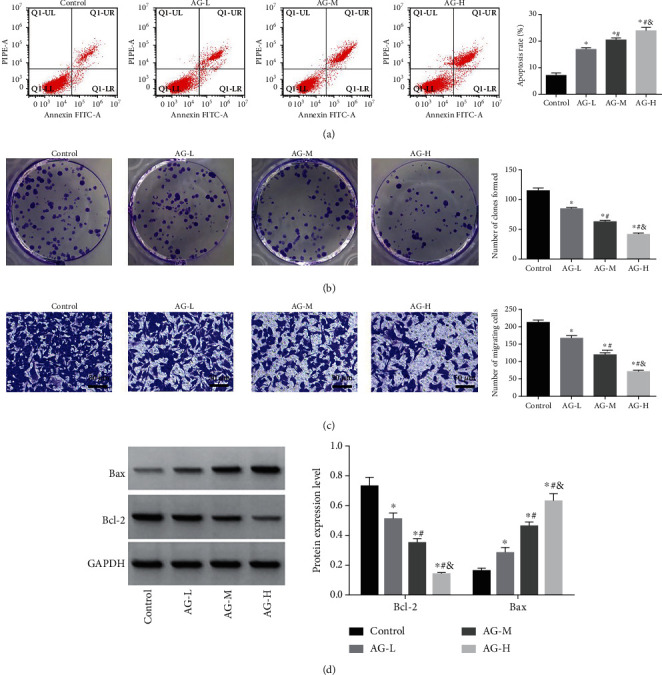
Impact of Amarogentin (AG) on HGC-27 apoptosis, cloning and migration a: Impact of AG on HGC-27 apoptosis. b: Impact of AG on HGC-27 clone number. c: Impact of AG on HGC-27 migration. d: Impact of AG on expression of Bax and Bcl-2 protein in HGC-27. ^∗^P <0.05 vs. control group; # P <0.05 vs. AG-L group; & P <0.05 vs. AG-M group.

Concentration-dependently, HGC-27 cells in AG-L, M, and H groups presented elevated cell multiplication inhibition rate, apoptosis and Bax protein level than control cells (P <0.05), with reduced number of cell clones formed and migrating cells as well as Bcl-2 protein (P <0.05), as shown in Figure 2 and Table 2.

### 3.3. AG's Impact on circKIF4A and miR-152-3p Expression in HGC-27

**Figure 3:**
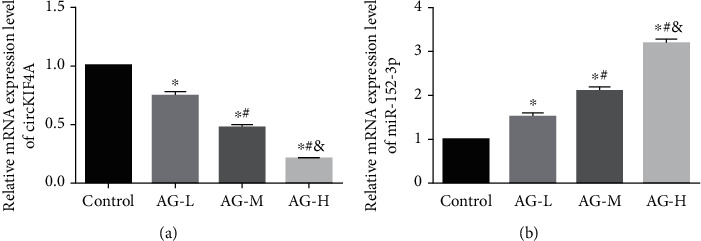
Impact of AG on circKIF4A and miR-152-3p expression in HGC-27 a: circKIF4A in HGC-27. b: miR-152-3p in HGC-27.^∗^ P <0.05 vs. control group; # P <0.05 vs. AG-L group; & P <0.05 vs. AG-M group.

Concentration-dependently, AG-L, M and H groups showed reduced circKIF4A (P <0.05) and elevated miR-152-3p (P <0.05) than the control group, as shown in Figure 3.

### 3.4. Impact of Inhibiting circKIF4A on HGC-27 Cell Multiplication, Migration and Apoptosis

**Figure 4:**
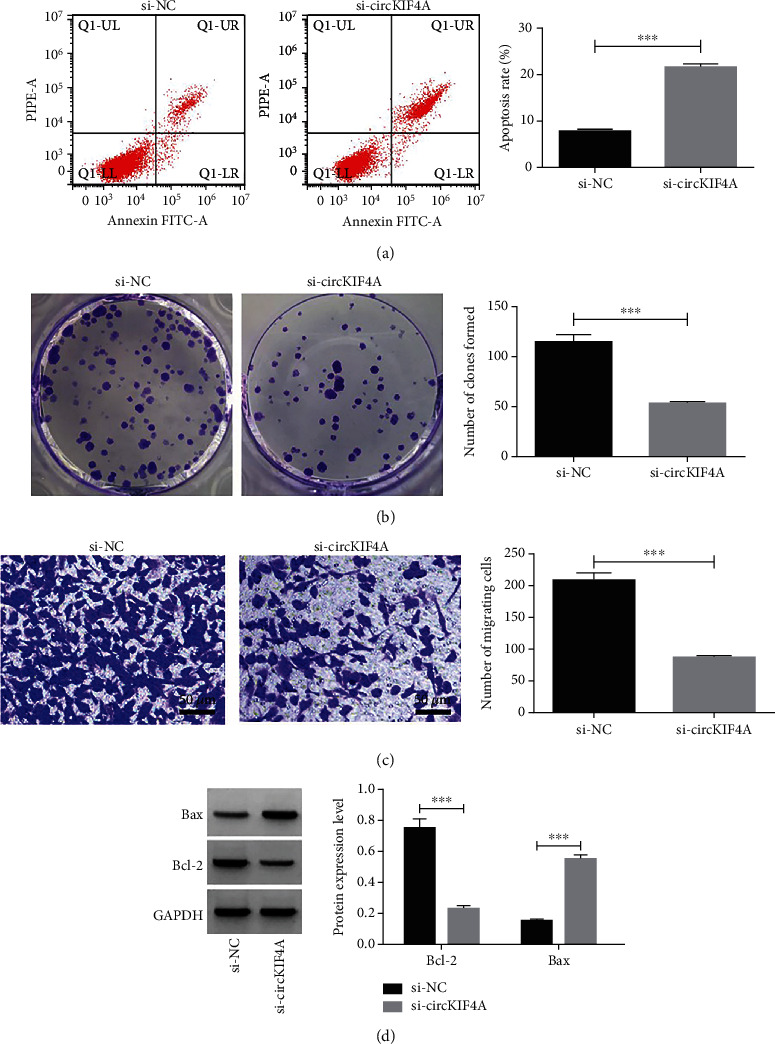
Impact of inhibiting circKIF4A on HGC-27 apoptosis, clone formation and migration a: Impact of inhibiting circKIF4A on HGC-27 apoptosis. b: Impact of inhibiting circKIF4A on HGC-27 clone formation number. c: Impact of inhibiting circKIF4A on HGC-27 migration. d: Impact of inhibiting circKIF4A onBax and Bcl-2 protein in HGC-27.^∗∗∗^P <0.001.

Increases in cell multiplication inhibition rate, apoptosis rate and Bax protein level (P <0.05), as well as reductions in cell clone formation number, migrating cell number, and Bcl-2 protein level (P <0.05) were observed in si-circKIF4A group versus si-NC group, as shown in Figure 4 and Table 3.

### 3.5. circKIF4A Targets miR-152-3p

**Figure 5:**
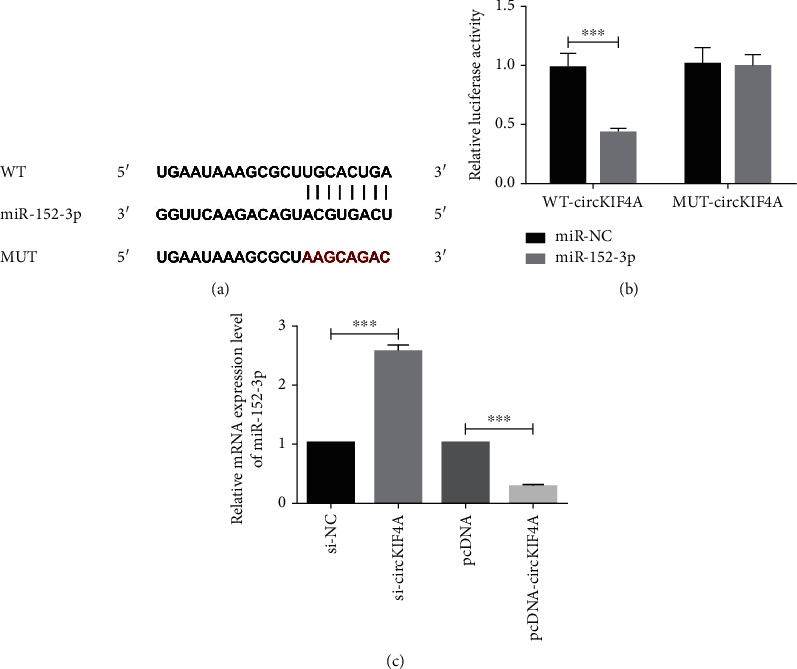
circKIF4A targets miR-152-3p a: Complementary sequences of miR-152-3p and circKIF4A. b: Double luciferase reporter assay. c: circKIF4A regulation of miR-152-3p. ^∗∗∗^ P <0.001.

circKIF4A and miR-152-3p have complementary sequences, as shown in Figure 5(a). Overexpressing miR-152-3p decreased wild-type vector WT-circKIF4A luciferase activity (P <0.05; Figure 5(b)), indicating the presence of targeted regulation between circKIF4A and miR-152-3p. miR-152-3p showed up-regulated levels in si-circKIF4A group versus si-NC group (P <0.05), and in pcDNA group versus pcDNA-circKIF4A group (P <0.05), as shown in Figure 5(c).

### 3.6. Impact of circKIF4A on AG-Intervened HGC-27 Multiplication, Migration and Apoptosis

**Figure 6:**
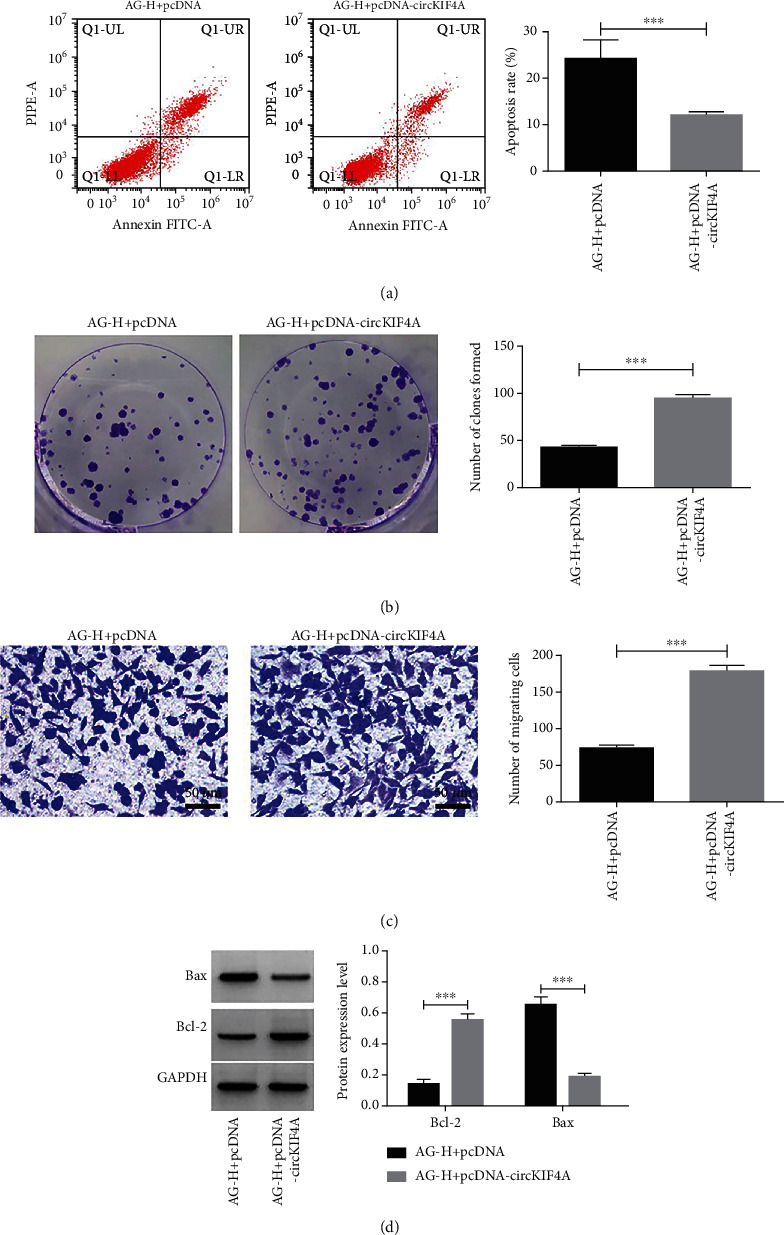
Impact of circKIF4A on Amarogentin (AG)-intervened HGC-27 cell apoptosis, clone formation and migration. a: Impact of circKIF4A on AG-intervened HGC-27 apoptosis. b: Impact of circKIF4A on AG-intervened HGC-27 clone formation number. c: Impact of circKIF4A on AG-intervened HGC-27 migration number. d: Impact of circKIF4A on Bax andBcl-2 protein in AG-intervened HGC-27. ^∗∗∗^ P <0.001.

In comparison with AG-H + pcDNA group, AG-H + pcDNA-circKIF4A group had decreased cell multiplication inhibition rate, apoptosis rate and Bax protein level (P <0.05), but increased cell clones formed, migrating cells, and Bcl-2 protein level (P <0.05), as shown in Figure 6 and Table 4.

## 4. Discussion

Some traditional Chinese medicines have anti-GC actions and can play an anti-GC role by modulating multiple genes and signal axises [20]. circRNAs are reported to be either up-regulated or down-regulated in GC, and can be miRNAs' sponge molecules and positively modulate their target genes' expression, thus regulating the biological behavior of GC cells. circRNAs are shown to play the role of oncogene or anti-oncogene in the genesis and development of GC, which may also be potential targets for targeted therapies of the disease [21, 22]. However, whether they can be candidate targets for traditional Chinese medicine treatment of GC has not been clarified.

AG belongs to secoiridiod glycosides with anti-tumor effects [10, 23], with the ability to inhibit angiogenesis of hepatoma cells [24]. However, its role in the biological behavior of GC cells remains unknown. This work showed increased multiplication inhibition rate of GC cells and reduced number of cell clones after AG treatment, suggesting that AG can inhibit GC cell multiplication and clone formation. Up-regulating Bcl-2 can suppress apoptosis, while increasing Bax expression promotes apoptosis [25]. This study revealed that with the increase of AG concentration, the apoptosis rate of GC cells and Bax protein increased, while Bcl-2 protein decreased, suggesting that AG can promote GC cell apoptosis. Besides, AG reduced the ability of GC cells to migrate, suggesting the suppression of AG on GC cell migration. Apoptosis, a primary conservative process of cell clearance, is an approach through which most anticancer drugs affect cancer cells, namely, by inducing the apoptosis network of carcinoma cells [26, 27]. Similar reports showed that in the mouse liver cancer model, AG induced liver cancer cell apoptosis via up regulating the ratio of Bax/Bcl-2, triggering the cleavage of Caspase-3 and poly adenosine diphosphate ribose polymerase [28].

circKIF4A is highly expressed in ovarian carcinoma and can promote cancer cell multiplication and metastasis [29]. It is also up-regulated in bladder cancer and can promote carcinoma cell metastasis [30]. Herein, circKIF4A in GC tissues and cells increased, and AG could concentration-dependently decrease circKIF4A levels, suggesting that AG might play an anti-GC role by inhibiting circKIF4A. Besides, we preliminarily confirmed that circKIF4A could target miR-152-3p. circRNAs have been reported to be miRNA sponges [31, 32]. In hepatocellular carcinoma, for example, circmto1 serves as miR-9's sponge to block tumor progression [33]. And circtp63 up regulates FoxM1 expression as ceRNA and promotes tumor progression in lung cancer [34]. This research demonstrates the ability of circKIF4A to interact directly with miR-152-3p. In GC cells, miR-152-3p was reportedly to be down-regulated, and up-regulating its level can inhibit cancer growth [35]. miR-152-3p overexpression can also suppress colorectal cancer cell migration and invasiveness [36]. This study identified decreased miR-152-3p in GC tissues and cells, which can be promoted by AG. Reductions in GC multiplication, clone formation, apoptosis and migration were observed after circKIF4A inhibition, while the overexpression of circKIF4A decreased AG's impacts on GC cell multiplication, clone formation, apoptosis and migration. All these suggest that AG can promote miR-152-3p expression via inhibiting circKIF4A, thus playing an anti-gastric cancer role.

This work still has limitations. The clinical implications of AG and circKIF4A in GC need further elucidation. Besides, it is necessary to find ideal tumor markers that can be applied to diagnose GC in the early stage. Moreover, the specific pathway involved in the primary mechanism by which AG suppresses GC cell multiplication and induces apoptosis deserves investigation. Thus, more experiments need to be conducted in the future, as well as more clinical research to find out the crucial role of circKIF4A in GC, and investigate the treatment value of AG.

## 5. Conclusion

To sum up, AG can inhibit GC cell multiplication, clone formation and migration, and promote apoptosis. circKIF4A is overexpressed while miR-152-3p is under-expressed in GC tissues and cells. AG can play an anti-GC role by suppressing circKIF4A to up-regulate miR-152-3p expression. Therefore, circKIF4A/miR-152-3p may serve as a candidate target of AG in treating GC. However, the specific mechanism of its action needs further exploration.

## Figures and Tables

**Table 1:** Sequences of primers.

Objects	Primer sequences
circKIF4A	Forward: 5'-GAGGTACCCTGCCTGGATCT-3'
	Reverse: 5'-TGGAATCTCTGTAGGGCACA-3'
miR-152-3p	Forward: 5'-CGCGTCAGTGCATGACAGA-3'
	Reverse: 5'-AGTGCAGGGTCCGAGGTATT-3'
U6	Forward: 5'-GCTTCGGCAGCACATATACTAAAAT-3'
	Reverse: 5'-CGCTTCAGAATTTGCGTGTCAT-3'
GAPDH	Forward: 5'-GGAGCGAGATCCCTCCAAAAT-3'
	Reverse: 5'-GGCTGTTGTCATACTTCTCATGG-3'

**Table 2:** Impacts of Amarogentin (AG) on HGC-27 multiplication, migration and apoptosis ($\overset{¯}{x}\pm s,n=9$).

Group	Control	AG-L	AG-M	AG-H	F	P
Inhibition rate/%	0.00 ± 0.00	21.64 ± 0.90^∗^	41.29 ± 1.90^∗^^#^	59.89 ± 2.69^∗^^#&^	2046.822	0.000

Note: ^∗^P <0.05 vs. control group; # P <0.05 vs. AG-L group; & P <0.05 vs. AG-M group.

**Table 3:** Detection of impacts of inhibiting circKIF4A on HGC-27 multiplication, migration and apoptosis ($\overset{¯}{x}\pm s,n=9$).

Group	circKIF4A	Inhibition rate/%
Si-NC	1.00 ± 0.00	0.00 ± 0.00
Si-circKIF4A	0.34 ± 0.03^∗^	52.89 ± 1.84^∗^
t	66.000	86.234
P	0.000	0.000

Note: ∗ P <0.05 vs. si-NC group.

**Table 4:** Detection of impacts of circKIF4A on Amarogentin (AG)-intervened HGC-27 multiplication, migration and apoptosis ($\overset{¯}{x}\pm s,n=9$).

Group	circKIF4A	Inhibition rate/%
AG-H + pcDNA	1.00 ± 0.00	59.43 ± 4.15
AG-H + pcDNA-circKIF4A	4.15 ± 0.13^∗^	15.15 ± 0.85^∗^
t	72.692	31.359
P	0.000	0.000

Note: ∗P <0.05 vs. AG-H + pcDNA group.

## Data Availability

The labeled dataset used to support the findings of this study are available from the corresponding author upon request.
